# The effect of various breathing exercises (pranayama) in patients with bronchial asthma of mild to moderate severity

**DOI:** 10.4103/0973-6131.53838

**Published:** 2009

**Authors:** Tarun Saxena, Manjari Saxena

**Affiliations:** Department of Internal Medicine, Swamy Consultant Physician Mittal Hospital, Ajmer, India; 1 PG Diploma in Yoga Science, MDS University, Ajmer, India

**Keywords:** Bronchial asthma, expiratory breathing exercises, FEV1, PEFR

## Abstract

**Background/Aim:**

The incidence of bronchial asthma is on increase. Chemotherapy is helpful during early course of the disease, but later on morbidity and mortality increases. The efficacy of yoga therapy though appreciated is yet to be defined and modified. Aim: To study the effect of breathing exercises (*pranayama*) in patients with bronchial asthma of mild to moderate severity.

**Materials and Methods:**

Fifty cases of bronchial asthma (Forced Expiratory Volume in one second (FEV1) > 70%) were studied for 12 weeks. Patients were allocated to two groups: group A and group B (control group). Patients in group A were treated with breathing exercises (deep breathing,*Brahmari*, and *Omkara*, etc.) for 20 minutes twice daily for a period of 12 weeks. Patients were trained to perform *Omkara* at high pitch (forceful) with prolonged exhalation as compared to normal *Omkara*. Group B was treated with meditation for 20 minutes twice daily for a period of 12 weeks. Subjective assessment, FEV1%, and Peak Expiratory Flow Rate (PEFR) were done in each case initially and after 12 weeks.

**Results:**

After 12 weeks, group A subjects had significant improvement in symptoms, FEV1, and PEFR as compared to group B subjects.

**Conclusion:**

Breathing exercises (*pranayama*), mainly expiratory exercises, improved lung function subjectively and objectively and should be regular part of therapy.

## INTRODUCTION

The incidence of bronchial asthma world over is on increase. The disease is characterized by[1] cough, wheezing, and breathlessness (expiratory difficulty). It is exacerbated by various factors like environmental factors, infections, occupational factors, cold exposure, exercises, etc. Presently, disease management strategy includes pharmacological therapy (inhaled/oral medicine). Initially this therapy is helpful in management of the disease, but later on there is increase in financial burden, morbidity (more and more patients requiring oxygen therapy/respiratory support therapy), and mortality.

Nonpharmacological therapy includes yogic techniques such as breathing exercises (*pranayama* – the basic vitality necessary to life is termed aspranaand regulation of *prana* is *pranayama*.), meditation, and asana.

Various studies have shown the effectiveness of these techniques in asthma,[2–4] hypertension,[5] diabetes, and ischemic heart disease,[6] but type, duration, and efficacy in asthma are not well established. Besides, modification of conventional exercises according to medical pathology has not been done yet; therefore, this trial was conducted.

## MATERIALS AND METHODS

The present study was conducted in the Department of Medicine, Mittal Hospital, Ajmer, India, in collaboration with The Department of Yoga Science, MDS University, Ajmer, India.

Fifty cases of bronchial asthma were chosen for the study following diagnostic confirmation. Symptoms of asthma, Forced Expiratory Volume in one second (FEV1) < 85%, and reversibility (increase in FEV1) > 12% after 20 minutes of two salbutamol puffs were used to confirm the diagnosis of bronchial asthma. The study cases had FEV1 > 70%, interest in yoga, and a minimum of six months experience in performing yogic practices.

Exclusion criteria were patients with symptoms suggestive of disease other than bronchial asthma like ischemic heart disease, bronchitis, and anemia and patients with history of smoking.

The test cases had no history of regular medication and they were advised to discontinue if on any medications.

Investigations done for the diagnosis of asthma were:

1. Routine medical checkup (pulse, BP, ECG)
2. Chest X-ray
3. FEV1[7] (by Medical International Research (MIR) spirometer)
4. Peak Expiratory Flow Rate (PEFR) (by mini Wright peak flowmeter)
5. Symptoms were recorded according to symptoms score.

Normalized technique was used. All tests including symptoms were reobserved after 12 weeks.

### Symptoms score

Symptoms were categorized into three types – cough, wheezing, and dyspnea – and scored as mild, moderate, and severe.

1. Cough – mild (< 5 minutes/day), moderate (5–10 minutes/day), severe (>10 minutes/day).
2. Wheezing – mild (did not disturb sleep at night or in daily routine), moderate (disturbed sleep or disturbed daily routine), severe (great distress at rest).
3. Dyspnea (breathlessness) – mild (breathless only on walking uphill, comfortable walking on ground level), moderate (breathless while walking on ground level), severe (breathless at rest).

None of the cases selected had severe symptoms.

Any reduction in these symptoms from moderate to mild or mild to absence was considered as improvement in the disease severity.

### Division of patients

Patients were randomly divided into two groups. Randomization was done by numbering the patients 1–50, even numbered patients were allocated to group A and odd numbered patients to group B. Both groups were comparable in all the aspects including age, sex, symptoms, and lung functions.

Group A (n= 25) practiced breathing exercises/pranyama for 20 minutes twice daily for 12 weeks.

Group B (n= 25, control group)practiced meditation for 20 minutes twice daily for 12 weeks.

Initial parameters of patients in both groups are given in Table 1.

**Table 1:** Initial parameters of patients

Parameters	Group A (exercise group)	Group B (control group)	*P* value
Number of patients	25	25	>.05
Men	12	13	>.05
Women	13	12	>.05
Mean age	29.1 ± 7.51 (18–45)	29.4 ± 7.58 (18–45)	>.05
Height (cm)	152–170	153–171	>.05
FEV1%	72 ± 1.70	73 ± 2.07	>.05
FEV1/FVC	72 ± 1.70	73 ± 2.07	>.05
Postbronchodilator change	12%	12%	>.05

### Breathing exercises/*pranayama* performed by group A[8]

1. Deep breathing (deep inspiration and deep expiration): subjects sit in *sukhasana* and perform deep inspiration and expiration through both nostrils.
2. *Sasankasana* breathing: subjects sit in *vajrasana* with their hands back, holding the right wrist with the left arm, with inhalation the person bends backward and with exhalation bends forward touching his/her forehead to the ground.
3. *Anuloma viloma*: common breathing practice, in which subjects breathe through alternate nostrils while sitting in *sukhasana*.
4. *Bhramari* chanting: sitting in *sukhasana* subjects inhale through both nostrils and while exhaling produce sound of female humming bee.
5. *Omkara* (modified): commonly used for meditation, but not included in regular breathing exercises, is an important exhalation exercise. Changes to this exercise, keeping in mind the asthmatic expiratory difficulty with air trapping, were made so as to strengthen expiration. Patients were advised to sit in *sukhasana* and to inhale deeply and then while exhaling produceOmkarawith maximum force and to continue until further exhalation is not possible.

During conventional *Omkara, Omkara* is pronounced as ooooo…mmm, but patients were advised to practice OOOOOOOOO…MMM (high pitch/forceful) with prolonged exhalation.

First three breathing practices were to normalize the breathing, while *Bhramari* and *Omkara* are expiratory exercises.

### Meditation[9] performed by group B

The control group patients practiced meditation in sitting posture with closed eyes. Patients were advised first to confirm the side of the nostril from wherein the air is coming maximum, then to concentrate on the same nostril, to appreciate the sound of air along with inward and outward movement of outer wall of nostril. Patients deeply concentrated (meditated) at the same point twice daily for 20 minutes.

## RESULTS

**Figure 1:**
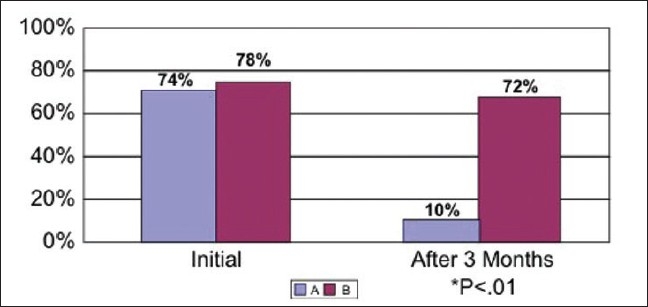
Symptoms in groups A and B, initially and after 12 weeks

**Figure 2:**
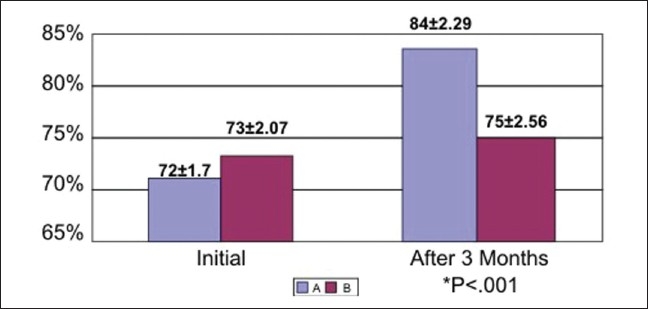
FEV1% in groups A and B, initially and after 12 weeks

**Figure 3:**
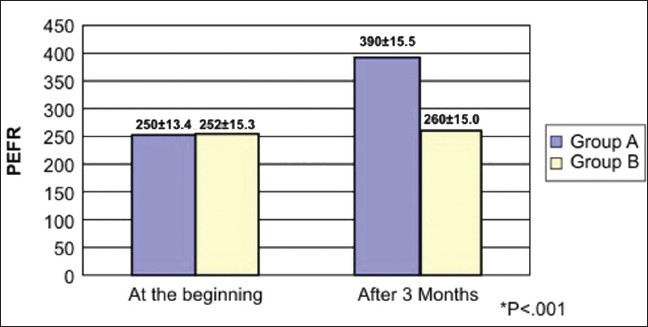
PEFR L/min in groups A and B after 12 weeks

After 12 weeks, there was significant reduction in symptoms [Table 2], improvement in FEV1 and PEFR in group A (*P*<.001) as compared to group B (control) [Figures 1–3]. Significance of symptoms was calculated by Mantel Haensal X^2^ formula.

**Table 2:** Pre-post symptom intensity after 12-week treatment

	Group A (exercise group) (%)	Group B (control group) (%)	*P* value
Cough			
Before	73	77	>.05
After 12 weeks	12	75	<.01
Wheezing			
Before	76	80	>.05
After 12 weeks	8	80	<.01
Dyspnea			
Before	73	77	>.05
After 12 weeks	10	79	<.01

Significance of FEV1% and PEFR was calculated by (mean1–mean2) divided by square root of (SD1^2^/n1+ SD2^2^/n2).

## DISCUSSION

Bronchial asthma, which has been increasing in incidence worldwide, is a morbid disease that can also be fatal. The important precipitating factors of asthma include occupational factors, viral infections, drugs, cold air, family history, stress, etc. It is a multifactor disease; clinically, it produces symptoms and signs like dyspnea (expiratory difficulty), cough, and wheezing. Pathologically, there is mucosal inflammation, collection of inflammatory mediators, bronchial constriction, air trapping, later on remodeling of airways. Presently, it is difficult to control all the triggers in a single patient. It is better to try to improve lung functions by exercises and to correct the pathology (common result to all the triggers); so stress was given to expiratory exercises[10] and some modification was done.

Fifty cases were selected with FEV1 > 70%. After confirming diagnosis, they were randomly divided into two groups, group A and group B. Group A performed breathing exercises and group B meditation. After 12 weeks there was significant improvement in symptoms and lung functions in group A.

This result is similar to other studies of Nagrathna *et al*., Goyeche *et al*., and McFadden[11] where improvement was found after yogic techniques. Reduction in psychosomatic factors was considered as prime factor in these studies, but improvement in our study was not due to any relaxation activity or decrease in psychosomatic factors because meditation group did not show any improvement. The results are also different from a few studies where some other techniques were used. In study of Cooper[12] *et al*. Butekyo breathing technique (a device which mimics *pranayama*) was used. There was minimal improvement in lung functions.

Study of Slader[13] included shallow nasal breathing with little improvement in lung functions. Similarly, study of Singh[14] used pink city lung exerciser with mild effectiveness.

**Figure 4:**
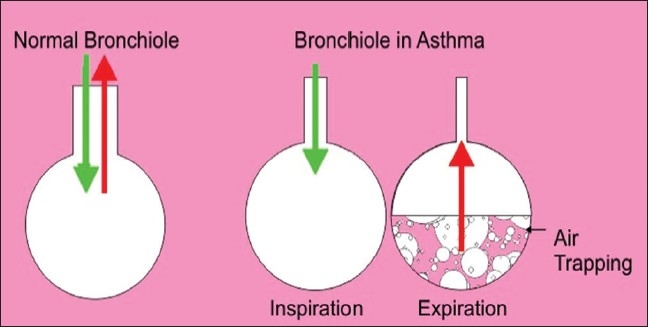
Forceful exhalation helps to open closed airways in asthma

**Figure 5:**
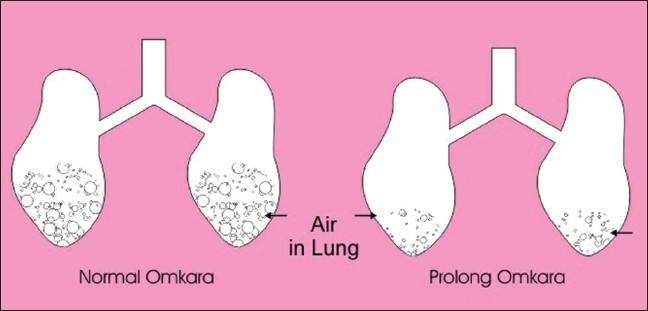
Prolonged exhalation helps to expel more trapped air in asthma

Three important things were found in the present study. Firstly, expiratory exercises are helpful. In bronchial asthma expiration is difficult, so exercises that support expiration are beneficial [Figures 4 and 5]. Secondly, forceful expiratory exercises (expiration) are helpful and this is illustrated in Figure 4. In this figure, air is easily coming in and going out in a normal person, but in an asthmatic patient air is coming ineasily but during expiration there is closing of airways[10] and force is required to open the airways; hence, a high pitch/forceful *Omkara* OOOOOOOOOO…MMMM was found to be helpful instead of oooo…mmm. Thirdly, prolonged expiratory exercises (expiration) are helpful. During normalOmkarathe air comes out only from the upper part of airways [Figure 5], but asthma is a disease that affects whole lower respiratory tract so prolonged exercise which helps to expire maximum trapped air is found beneficial [Figure 5]. Normally, Omkarais stopped after 10–15 seconds like oooooo…mmm, but the beneficial effect in asthma is found with prolonged OOOOOOOOOOOOO…MMMM until further expiration is not possible.
